# Hot Water Tuber Treatments for Management of *Meloidogyne Arenaria* in Caladium Cultivars

**DOI:** 10.2478/jofnem-2022-0016

**Published:** 2022-06-15

**Authors:** Mengyi Gu, Robert D. Hartman, Johan A. Desaeger

**Affiliations:** 1 Department of Entomology and Nematology, Gulf Coast Research and Education Center, University of Florida, Wimauma, FL 33598 United States of America; 2 Classic Caladium, LLC, Avon Park, FL 33825 United States of America

**Keywords:** Caladium, *Caladium* × *hortulanum*, hot water treatment, management, *Meloidogyne arenaria*, non-chemical, root-knot nematodes, thermotherapy

## Abstract

Hot water treatment was evaluated for its efficacy in controlling *Meloidogyne arenaria* on caladium. Caladium tubers pre-infested with *M. arenaria* were immersed in hot water at 50°C for 0 min, 30 min, and 45 min before being planted into 16.5-cm pots filled with sterilized sandy soil. Two caladium cultivars Florida Sweetheart PP 8526 (SWT) and Postman Joyner (PJR), each with three tuber sizes [#3 (<1.5 inch), #1 (1.5–2.5 inch), and Jumbo (>2.5 inch)], were evaluated. Ninety days after the first shoot observation, the number of nematode eggs in roots and second-stage juveniles in soil were significantly reduced, but not eliminated, in both 30 min and 45 min treatments; the 45 min treatment had better results than the 30 min treatment. The efficacy of hot water treatment was affected by caladium cultivar, but not by tuber size. The treated PJR tubers had lower nematode numbers than those of the cultivar SWT. The difference in nematode number between the two caladium cultivars might be related to the morphological characteristics of caladium tubers, as the scale-like tissue on SWT tubers might provide refuge for root-knot nematodes from heat damage. Further research needs to be conducted on determining heat-tolerant thresholds for different *Meloidogyne* spp. and different caladium cultivars, which will help improve nematode management strategies for caladium growers.

Caladiums (*Caladium* ⨯ *hortulanum*) are popular leafy ornamental crops native to the tropical regions of America. Highlands County, Florida has been leading the commercial production of caladium tubers by providing over 95% of the world’s tuber supply (Kokalis-Burelle et al., 2010; Deng, 2018). In Florida, caladium tubers are produced both in organic (“muck”) and sandy soils, with the latter providing the most favorable environment for root-knot nematode reproduction (Kim et al., 2017). The 9-mon growing season (from planting to tuber harvest) of caladiums provides root-knot nematodes ample opportunity to build up their population density, causing severe plant damage, and resulting in a decline in yield and quality of tubers. Nematode-infested caladium tubers have an impact on both retail sales and replanting, which leads to economic losses (Gu and Desaeger, 2021). Above ground root-knot nematode symptoms have an irregular distribution in caladium fields and include leaf dieback, plant stunting, wilting, and yellowing. Below ground symptoms are more diagnostic and include galled roots and tubers, root and tuber rot, and corky lesions on tubers (Kokalis-Burelle et al., 2017).

Currently, no root-knot nematode resistant caladium cultivars or post-plant nematicides are available for caladium growers. Biological products including live organisms such as different *Bacillus* spp., *Trichoderma spp.,* and *Purpureocillium lilicanus*, as well as natural toxins from *Burkholderia*, azadirachtin, and thyme oil, are available, but no information indicating their efficacy in controlling root-knot nematodes in caladium is available. Pre-planting soil fumigation is the most widely adopted nematode management strategy by Florida caladium growers (Gilreath et al., 1999; Rosskopf et al., 2005; Kokalis-Burelle et al., 2010; Gu and Desaeger, 2021). However, soil fumigation will not protect against nematodes that are introduced with planting material. Fumigation may worsen the damage in that case as its broad-spectrum activity can create a biological vacuum and loss of natural biocontrol, which may increase root-knot damage when nematodes are introduced through nematode infected caladium tubers (Yakabe et al., 2010). Therefore, introducing nematode-free plant material is one of the most important steps in nematode management for caladium (Khanal et al., 2020).

Thermotherapy, as a non-chemical and environmentally friendly method, has been applied for plant disease management since the 1930s through hot water, air, and vapor (Kunkel, 1935, 1936). The first hot water treatment study on controlling root-knot nematodes in caladium tubers was conducted in 1961 (Rhoades, 1964). Based on the results, pre-planting hot water treatment at 50°C for 30 min has been adopted in the caladium tuber production industry as a standard (Kokalis-Burelle et al., 2010). The main objectives of this study are (1) to investigate the efficacy of different hot water immersion times, and (2) to evaluate if hot water treatment efficacy is affected by caladium variety and tuber size.

## Materials and Methods

### Greenhouse assay

Greenhouse studies were conducted at the University of Florida Gulf Coast Research and Education Center (UF/GCREC), Wimauma, Florida between February and June 2020. Experiments were established in 16.5-cm diameter × 14.6-cm tall plastic pots with a soil holding capacity of 2 kg. The soil (95% sand, 2% silt, 3% clay with 0.9% organic matter, A & L Western Laboratories, Inc., CA) was heat sterilized at 185°F for 2 hr. During the study period, plants were maintained in a greenhouse with a temperature = 24 ± 3°C and humidity = 64 ± 12%. Caladium tubers were obtained from a commercial caladium field and were naturally infested with the peanut root-knot nematode, *Meloidogyne arenaria*. Two caladium cultivars Florida Sweetheart PP 8526 (SWT) and Postman Joyner (PJR), each with three tuber sizes [“#3” (<1.5 inch), “#1” (1.5–2.5 inch), and “Jumbo” (> 2.5 inch)], were used in this experiment. Tubers were immersed in a Chattanooga M-4 Hydrocollator^®^ hot pack heater (DJO, LLC, Vista, CA) for 0 min, 30 min, and 45 min at 50°C. After drying overnight, treated and non-treated tubers were dusted with dolomite and *Trichoderma* spp. (RootShield *Plus***^+^** WP, BioWorks, Victor, NY) at a ratio of 16:1 before planting to prevent tuber rotting. Pots were arranged in a randomized complete block design with five replications. The experiment was repeated once, with a 1-wk interval between the two experiments. Plants were watered as needed and fertilized biweekly with 50 mL/pot of a 1.85 g/L solution of Miracle-Gro fertilizer (Scotts Miracle-Gro Company, Marysville, OH).

Both experiments were terminated 90 d after the observation of the first shoot. Root-knot nematode eggs were extracted by shaking the whole root system of each plant in a 0.6% sodium hypochlorite solution for 2 min (Hussey and Barker, 1973). Second-stage juveniles (J2) were extracted from a well-mixed 200 cm^3^ subsample of soil from each pot using a modified Baermann funnel method with 2-d incubation at 25°C (Forge and Kimpinski, 2007). Plant heights were collected from each plant. Fresh shoots and roots from each pot were weighed separately, then dried at 54°C for a week and weighed again.

### Data analysis

Data were analyzed using the Glimmix model in SAS University Edition (SAS Institute, Cary, NC). Data from both repetitions were combined if no statistically significant differences were found between experiments (*P >* 0.05). Nematode egg numbers per root system or per unit of fresh or dry root weight, as well as juvenile soil population data, were fit to Lognormal distribution, and data on gall rating and plant health parameters were fit to Gaussian distribution. Non-transformed least squares means (LSMeans) were used in tables and figures and LSMeans of data were separated by Tukey–Kramer Test (*P* ≤ 0.05).

## Results

*M. arenaria* reproduction was significantly affected by hot water exposure time and caladium cultivar (Table 1). Both 30 min and 45 min hot water exposure statistically (*P <* 0.01) reduced gall rating, numbers of nematode egg per root system, per gram fresh root and per gram dry root, and J2 numbers in the soil. Nematode-infested caladium tubers treated with 45 min hot water exposure had fewer gall symptoms, and egg and J2 numbers, than these treated with 30 min exposure. More root-knot nematode eggs were extracted from roots of cultivar SWT than those of PJR (*P <* 0.01). On the other hand, fewer J2s were observed in SWT soil than in PJR soil (*P =* 0.04). Hot water exposure time and caladium cultivar showed a significant interaction for root gall rating (*P =* 0.05), egg numbers per root system (*P <* 0.01), and J2 numbers in soil (*P <* 0.01). Tuber size did not have effect on any of the *M. arenaria* infection or reproduction results.

**Table 1:** An ANOVA summary table. Effects of hot water exposure time, caladium cultivar, and tuber size on tuber infection and reproduction of root-knot nematode (*Meloidogyne arenaria*).

Factor		Gall index^a^		Eggs per			Eggs per			Eggs per			Number of J2		
		(0–10)		fresh root system^a^			gram fresh root^a^			gram dry root^a^			per 200 cc soil^a^		
	DF	*F*	P-value	DF	*F*	*P*-value	DF	*F*	*P*-value	DF	*F*	*P*-value	DF	*F*	*P*-value
Exposure time	2	247.12	<0.01	2	242.76	<0.01	2	304.14	<0.01	2	260.12	<0.01	2	274.5	<0.01
Cultivar	1	0.43	0.51	1	11.07	<0.01	1	13.10	<0.01	1	13.67	<0.01	1	4.20	0.04
Tuber size	2	1.35	0.26	2	0.27	0.77	2	0.06	0.94	2	0.00	1.00	2	0.21	0.81
Exposure time × Cultivar	2	3.07	0.05	2	8.81	<0.01	2	6.83	<0.01	2	7.99	<0.01	2	5.61	<0.01
Exposure time x Tuber size	4	1.87	0.12	4	1.33	0.26	4	1.02	0.40	4	1.37	0.25	4	1.33	0.26
Cultivar × Tuber size	2	4.33	0.01	2	0.59	0.55	2	0.58	0.56	2	0.67	0.52	2	0.13	0.88
Exposure time x Cultivar × Tuber size	4	2.30	0.06	4	0.71	0.59	4	0.46	0.76	4	0.66	0.62	4	0.32	0.86

a Data were fit to Lognormal distribution in the Glimmix model in SAS University Edition.

The effects of different factor combinations on the reproduction of *M. arenaria* are presented in Table 2. Few root galls were observed following 30 min and 45 min hot water exposure with both cultivars. Root-knot egg numbers ranged from 0 to 78,855 per root system, 0 to 6,418 per gram fresh root, and 0 to 201,141 per gram dry root, with #3 PJR exposed for 45 min and #1 SWT exposed for 45 min supporting the lowest numbers, and #3 SWT exposed for 30 min supporting the highest numbers. Both 30 min and 45 min hot water exposure reduced J2 numbers in soil, and fewer nematodes were recovered from the soil of PJR than that of SWT after receiving hot water treatments.

**Table 2:** Effects of factor combinations (hot water exposure time, caladium cultivar, and tuber size) on reproduction of root-knot nematode (*Meloidogyne arenaria*) on caladium.

Cultivar	Exposure time @ 50°C (min)	Tuber size	Gall index (0–10)	Eggs per fresh root system^‡^	Eggs per gram fresh root^‡^	Eggs per gram dry root^‡^	Number of J2 per 200 cc soil^‡^
PJR	45	#3	0.0 c^†^	0 e	0 c	0 f	23 b
		#1	0.2 c	30 de	2 c	37 ef	23 b
		Jumbo	0.2 c	30 de	2 c	37 def	0 b
	30	#3	0.0 c	30 de	3 c	96 def	0 b
		#1	0.0 c	90 de	6 c	105 def	0 b
		Jumbo	0.0 c	270 de	16 c	351 def	35 b
	0	#3	4.2 b	218,325 ab	18,008 a	526,917 a	28,500 a
		#1	5.5 ab	297,060 a	15,950 a	336,192 ab	40,486 a
		Jumbo	7.5 a	441,465 a	24,584 a	465,989 a	18,169 a
SWT	45	#3	0.7 c	47,265 de	3,418 bc	128,769 def	1,563 b
		#1	0.0 c	0 e	0 c	0 f	0 b
		Jumbo	0.0 c	270 de	13 c	364 def	0 b
	30	#3	0.9 c	78,855 cd	6,418 bc	201,141 cd	2,218 b
		#1	0.7 c	37,320 bc	1,977 b	53,004 bc	3,838 b
		Jumbo	0.9 c	75,840 cd	2,725 bc	49,099 cde	7,010 b
	0	#3	5.6 ab	275,415 a	22,800 a	731,665 a	11,940 a
		#1	5.0 b	245,490 ab	19,077 a	497,953 ab	15,698 a
		Jumbo	5.1 b	343,875 a	21,171 a	517,797 a	23,823 a

† Within each column, LSMeans sharing the same letter are not significantly different (*P >* 0.05), according to Tukey– Kramer Test. **^‡^**Data were fit to Lognormal distribution in the Glimmix model in SAS University Edition, and non-transformed data were presented. LSMeans, least squares means; PJR, Postman Joyner; SWT, Florida Sweetheart PP 8526.

Hot water exposure time (*P <* 0.01) statistically affected caladium shoot and root growth (Table 3).

**Table 3:** An ANOVA summary table. Effects of hot water exposure time, caladium cultivar, and tuber size on caladium growth parameters.

Factor	Fresh shoot weight^†^ (g)			Dry shoot weight^†^ (g)			Fresh root weight^†^ (g)			Dry root weight^†^ (g)			Plant height^†^ (cm)		
	DF	*F*	*P*-value	DF	*F*	*P*-value	DF	*F*	*P*-value	DF	*F*	*P*-value	DF	*F*	*P*-value
Exposure time	2	19.62	<0.01	2	8.03	<0.01	2	18.46	<0.01	2	6.07	<0.01	2	0.58	0.56
Cultivar	1	9.75	<0.01	1	29.47	<0.01	1	0.03	0.87	1	5.37	0.02	1	389.94	<0.01
Tuber size	2	275.65	<0.01	2	181.33	<0.01	2	13.83	<0.01	2	32.07	<0.01	2	12.28	<0.01
Exposure time × Cultivar	2	1.86	0.16	2	0.94	0.39	2	6.08	<0.01	2	6.98	<0.01	2	6.50	<0.01
Exposure time x Tuber size	4	0.87	0.49	4	0.49	0.75	4	0.87	0.48	4	0.47	0.76	4	1.16	0.33
Cultivar × Tuber size	2	4.63	0.01	2	4.57	0.01	2	2.12	0.12	2	3.34	0.04	2	1.06	0.35
Exposure time x Cultivar × Tuber size	4	1.73	0.15	4	1.83	0.13	4	0.88	0.48	4	0.56	0.69	4	1.21	0.31

† Data were fit to Gaussian distribution in the Glimmix model in SAS University Edition.

Both 30 min and 45 min hot water exposure similarly improved caladium shoot and root growth; root weight was greater when caladium tubers received 45 min hot water treatment as compared to 30 min treatment. Caladium cultivar PJR had greater (*P <* 0.05) fresh and dry shoot weights, dry root weights, and plant height than cultivar SWT (Table 3). Caladium plant growth was significantly (*P <* 0.01) related to tuber size, with larger tubers having higher shoot and root weights, and plant height (Table 3). Interactions were noted between hot water exposure time and caladium cultivar (*P <* 0.05) for caladium root growth and plant height, and between caladium cultivar and tuber size (*P <* 0.05) for caladium shoot and root growth (Table 3).

**Figure 1:**
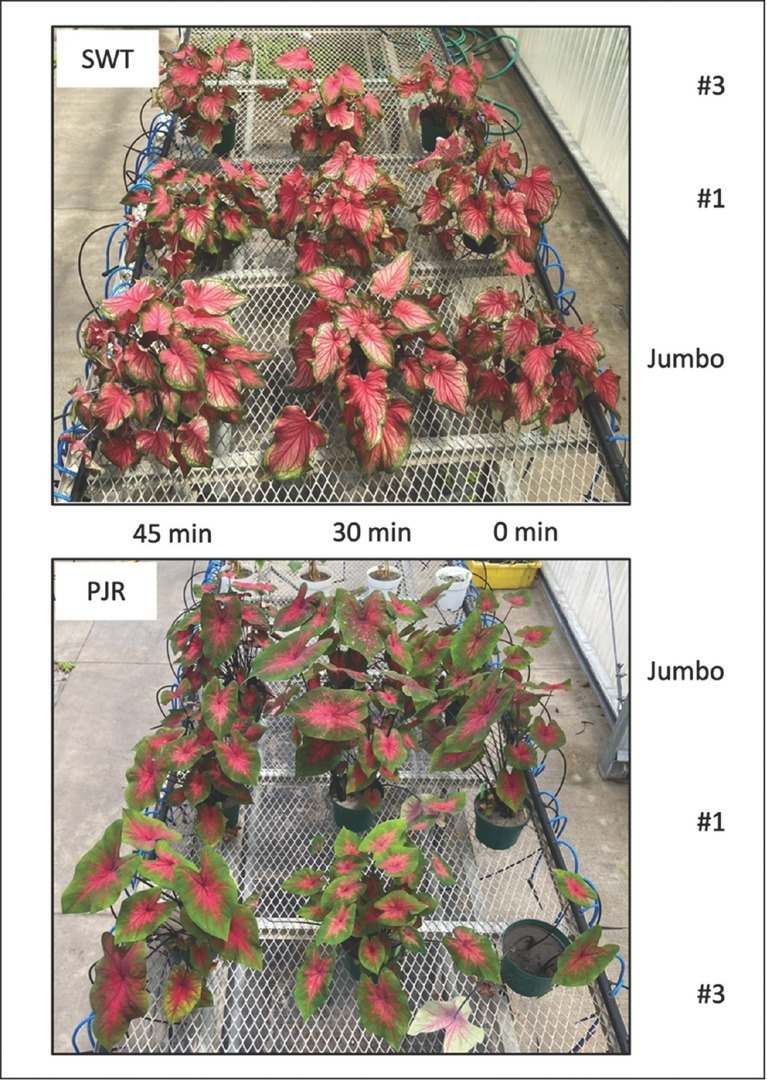
Aboveground symptoms of the two caladium cultivars SWT and PJR, each with three tuber sizes “#3” (small), “#1” (medium), and “Jumbo” (large), at the end of the experiment. Within the same tuber size, 30-min and 45-min hot water treated caladium tubers had more shoots when compared to the control. PJR, Postman Joyner; SWT, Florida Sweetheart PP 8526.

The effects of different factor combinations on caladium plant growth are presented in Table 4. Fresh shoot weight ranged from 67.8 g to 197.1 g, with #3 PJR exposed for 0 min and Jumbo PJR exposed for 30 min supporting the lowest and highest numbers; dry shoot weight ranged from 4.4 g to 13.1 g with #3 SWT exposed for 0 min and Jumbo PJR exposed for 30 min supporting the lowest and highest weights; fresh root weight ranged from 10.0 g to 24.0 g with #1 SWT exposed for 0 min and Jumbo SWT exposed for 45 min supporting the lowest and highest weights; dry root weight ranged from 0.33 g to 1.02 g with #3 SWT exposed for 0 min and Jumbo SWT exposed for 45 min supporting the lowest and highest numbers. Similar trends were observed for caladium shoot and root weights, with the greater shoot and root weights resulting from larger tubers for the same condition of hot water treatment, regardless of caladium cultivar. For both caladium cultivars, within the same tuber size, hot water treated caladium tubers had more shoots and roots when compared to non-treated caladium tubers (Fig. 1). Overall, cultivar PJR grew taller than cultivar SWT.

**Table 4:** Effects of factor combinations (hot water exposure time, caladium cultivar, and tuber size) on caladium growth parameters.

Cultivar	Exposure time @ 50°C (min)	Tuber size	Fresh shoot weight (g)	Dry shoot weight (g)	Fresh root weight (g)	Dry root weight (g)	Plant height (cm)
PJR	45	#3	89.8 ghij^†^	6.4 fghij	15.5 bc	0.59 abc	34.4 b
		#1	121.0 efg	8.0 defg	20.2 ab	0.93 ab	34.3 b
		Jumbo	184.2 ab	11.7 ab	20.9 ab	0.90 ab	36.3 ab
	30	#3	74.7 ij	5.4 hij	13.85 bc	0.5 bc	33.8 b
		#1	131.0 def	9.3 bcde	14.8 bc	0.75 abc	36.0 ab
		Jumbo	197.1 a	13.1 a	17.1 abc	0.85 ab	39.8 a
	0	#3	67.8 j	5.1 ij	13.1 bc	0.52 bc	34.2 b
		#1	106.5 fghi	8.1 defg	18.3 abc	0.93 ab	37.7 ab
		Jumbo	154.7 bcd	11.3 abc	15.3 bc	0.88 ab	37.4 ab
SWT	45	#3	84.0 hij	5.2 ij	15.6 bc	0.50 bc	27.5 c
		#1	126.9 def	7.9 efgh	21.4 ab	0.84 ab	27.6 c
		Jumbo	169.3 abc	10.5 bcd	24.0 a	1.02 a	28.4 c
	30	#3	80.7 hij	5.7 ghij	15.4 bc	0.54 bc	25.1 c
		#1	111.8 efgh	7.6 efghi	17.4 abc	0.69 abc	25.7 c
		Jumbo	155.2 bcd	10.0 bcde	20.8 ab	1.00 a	27.4 c
	0	#3	67.9 j	4.4 j	10.1 c	0.33 c	23.9 c
		#1	99.3 fghij	6.7 fghij	10.0 c	0.39 c	24.3 c
		Jumbo	143.5 cde	8.9 cdef	15.5 bc	0.69 abc	27.3 c

† Within each column, LSMeans sharing the same letter are not significantly different (*P >* 0.05), according to Tukey– Kramer Test. LSMeans, least squares means; PJR, Postman Joyner; SWT, Florida Sweetheart PP 8526.

## Discussion

Root-knot nematodes are the most widely distributed plant-parasitic nematodes in the world. Caladium is very susceptible to five species of root-knot nematode that have been reported in Florida (Kokalis-Burelle et al., 2017). Hot water treatment has been used for more than 100 yr across the world as an environmentally friendly pre-planting disease management strategy, and included in the purposes for which it is applied is control of plant-parasitic nematodes on different crops, such as bananas, rice, citrus, vegetable tubers, and ornamentals (Bridge, 1975). While different nematode genera have different heat-tolerant thresholds (Khanal et al., 2020), limited heat threshold information is available for species within the same nematode genus (Rhoades, 1961; Brcka et al., 2000).

**Figure 2:**
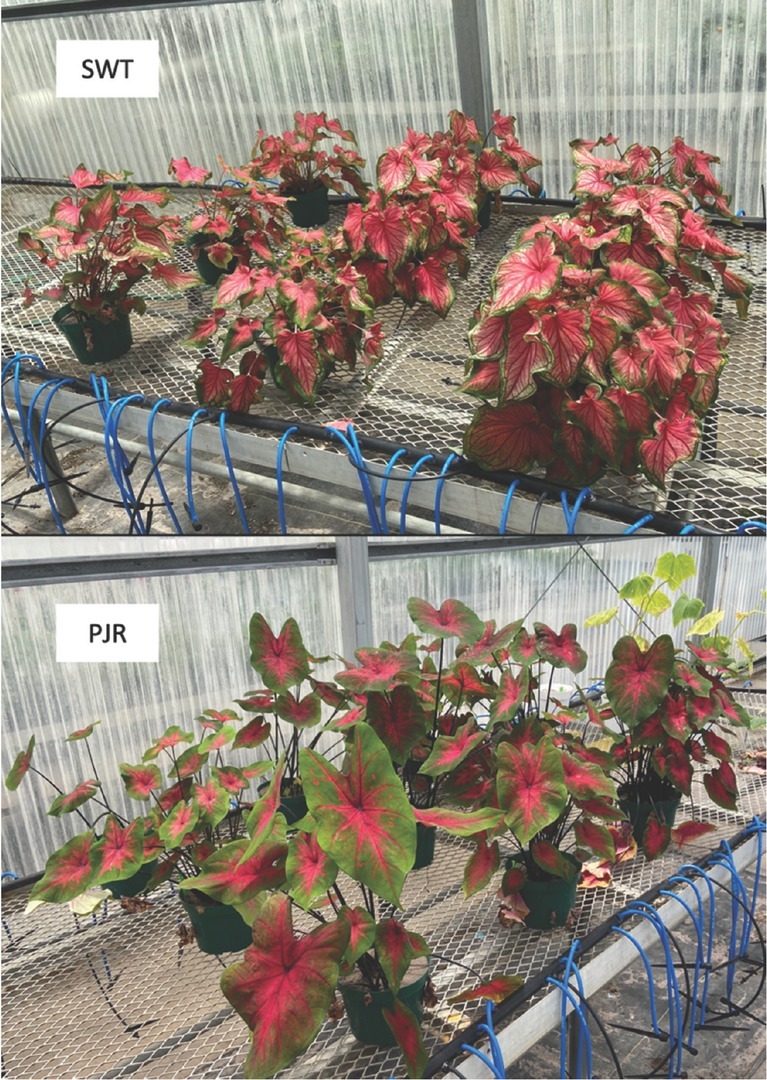
The appearances of the two caladium cultivars SWT and PJR. Cultivar PJR grows taller and has bigger leaves than cultivar SWT. PJR, Postman Joyner; SWT, Florida Sweetheart PP 8526.

The results from our study confirm that hot water treatment significantly reduces root-knot nematode population density in caladium tubers, and can significantly reduce root-knot damage caused by planting infected tubers (Rhoades, 1970; Brcka *et al*, 2000; Kokalis-Burelle, 2010). This was demonstrated by the severe growth reduction that was observed in our experiment when nematode-infested caladium tubers did not receive hot water treatment. Our data showed that while 45-min immersion times were more effective than 30-min exposure, neither 30-min nor 45-min hot water immersion at 50°C were able to completely eliminate *Meloidogyne arenaria* in caladium tubers. The efficacy of hot water treatment was also significantly affected by the caladium cultivar, which might be due to the morphological characteristics of different cultivars. Unlike tubers of cultivar PJR, which were mostly smooth, tubers from cultivar SWT were often covered by scale-like tissue, which might protect root-knot nematodes from heat stress. More testing on other caladium cultivars is needed to confirm if this is true or if other mechanisms are at play, and whether hot water affects other cultivars differently. Caladium plant growth was related to cultivar, with the cultivar PJR growing more vigorously than the cultivar SWT (Fig. 2); also, tuber size significantly affected caladium growth, with larger tubers (having more buds or eyes) producing higher leaf numbers and greater aboveground biomass (Deng, 2018).

Although root-knot nematodes in caladium tubers were not eliminated by hot water treatments, the previous results confirm the benefits of hot water treatment for the management of root-knot in caladium. A longer exposure time (45 min instead of the industry standard of 30 min) may be needed, especially when tubers are more infected, and for cultivars such as SWT that have more scale-like tissue. Tuber size did not seem to affect hot water efficacy, indicating that the nematode contamination is near the surface of the tubers, and no differential treatments are needed to treat different tuber sizes. Elimination of root-knot nematodes from caladium tubers will require increasing hot water immersion temperature or exposure time. While hot water treatment at higher temperatures delayed caladium tuber germination (Rhoades, 1961), it is not known how this affects subsequent growth in the field, and whether or not longer exposure times can be a safe option. In conclusion, hot water treatments remain an essential tool for caladium growers to help manage nematode and disease problems.
